# Studies on the mechanisms of bile acid initiated hepatic inflammation in cholestatic liver injury

**Published:** 2017-06-19

**Authors:** Shi-Ying Cai, James L. Boyer

**Affiliations:** Department of Internal Medicine and Liver Center, Yale University School of Medicine, New Haven, CT 06520, USA

**Keywords:** Bile acids, inflammatory cytokines, cholestasis

## Abstract

The mechanism of bile acid induced cholestatic liver injury remains controversial, thus hindering the development of new therapies for these diseases. In this research highlight, we briefly review the evolution of our understanding of the pathogenesis of bile acid induced liver injury, and summarize our recent findings on this topic. Our data suggests that under pathophysiological conditions bile acid induced liver injury is mediated by inflammatory responses that are initiated from stressed hepatocytes. We conclude by mentioning potential new therapeutic approaches for treating cholestatic liver injury based on these pathophysiologic concepts.

Chronic cholestasis, whatever the cause, results in liver injury where elevated hepatic levels of bile acid contribute to the exacerbation of the disease. However, the mechanism remains controversial. Earlier concepts held that high hepatic levels of bile acids killed hepatocytes through their direct cytolytic effects ^[1–3]^. In these studies, cultured human hepatocytes were exposed to submillimolar levels of toxic secondary bile acids. However, the concentration of these bile acid species in patient serum rarely reached these experimental levels ^[4]^. Subsequent studies proposed that the cause of cell death in cholestasis was due to bile acid induced hepatocyte apoptosis ^[5]^. This hypothesis was primarily based on observations in cultured rat hepatocytes treated with high levels (≥50 μM) of glycodeoxycholic acid or glycochenodeoxycholic acid (GCDCA) ^[6–8]^. However 1) GCDCA is not a major endogenous bile acid in rodents, and its concentrations in serum and liver are less than 10 μM even in bile duct ligated rats ^[9]^; and 2) no apoptotic cells have been detected in cholestatic livers ^[10–12]^. In contrast, taurocholic acid (TCA), the major endogenous bile acid in rodents, did not cause apoptosis in these cells even at millimolar concentrations ^[7]^. Recently, Allen *et al* showed that TCA treatment of cultured mouse hepatocytes (200 μM for 6 h) stimulated chemokine expression ^[13]^. These authors proposed that bile acid induced liver injury was mediated via an acute inflammatory response. This hypothesis is also supported by finding neutrophil infiltrations in necrotic areas of liver following bile duct ligation in rodents ^[14]^. They also found that the bile acid induction of chemokines is FXR independent but partially Egr1-dependent ^[13]^. Since Egr1 is a transcription factor expressed in many cells and tissues, they speculated that bile acid activation of Egr1 was mediated through a membrane receptor although the nature of this signaling pathway remained elusive ^[13]^. In addition, the functional significance of bile acid induced chemokine production in liver injury remained to be explained.

Our interest in the mechanism of bile acid induced liver injury came about by observations we obtained concerning the evolution and progression of cholestatic liver injury in the *Mdr2* (*Abcb4*)^−/−^ mouse ^[15, 16]^. In those studies, we sequentially assessed the development of liver injury over time in 10-day, 3-week, 6-week and 12-week old *Mdr2*^−/−^ mice by examining liver histology, gene expression, neutrophil infiltration, and plasma levels of bile acids at these various time points compared to their littermate wild-type controls. Plasma levels of bile acids in these *Mdr2*^−/−^ mice were significantly higher than in the wild-type controls at all time periods measured after birth and rosein parallel with the increased expression in the liver of proinflammatory cytokine mRNAs for Tnfα, Ccl2, Cxcl1, and Cxcl2. In 10-day old mice, the number of hepatic neutrophils were similar between *Mdr2*^−/−^ and wild-type control mice. However, marked elevations in neutrophil infiltrations were first detected in the 3-week *Mdr2*^−/−^ livers, while histological evidence of liver injury did not become apparent until these *Mdr2*^−/−^ mice were 6-weeks of age. This chronological data clearly indicated that a hepatic inflammatory response precedes in time the development of liver injury, thus directly supporting the concept that it is the cytokine induced inflammatory response that first results in cholestatic liver injury.

In our recent study, summarized below, we more directly assessed the role of proinflammatory chemokines in cholestatic liver injury ^[12]^. First, we challenged *Ccl2*^−/−^ mice and their wild-type controls with 1% cholic acid in their diet for 7 days. Significantly lower levels of plasma bile acids and alanine aminotransferase (ALT) were found in *Ccl2*^−/−^ livers than in the wild-type controls, suggesting that deletion of the chemokine Ccl2 protects mice from cholestatic liver injury presumably by muting the inflammatory response. To confirm these results, we examined the effects of 7-day bile duct ligation in these *Ccl2*^−/−^ mice. Assessment of liver histology revealed marked necrosis in the wild-type livers, that was nearly absent in *Ccl2*^−/−^ livers. Plasma ALT in wild-type mice was 3 times the levels seen in *Ccl2*^−/−^ mice, despite similar levels of plasma bile acids in both genotypes. FACS analysis and immunohistochemistry demonstrated significantly less infiltrates of neutrophils and T-cells but not macrophages in the livers from *Ccl2*^−/−^ mice when compared to the wild-type control. Together, these findings confirmed that inflammatory chemokines play a key role in bile acid induced liver injury where neutrophils and T-cells may mediate these events.

To further investigate the mechanism of this bile acid induced inflammatory response in the liver, we first isolated liver parenchymal and non-parenchymal cells from the mice and treated them with the major species of endogenous bile acids for various times. Only hepatocytes were induced to stimulate the expression of proinflammatory chemokines (Ccl2, Cxcl1 and Cxcl2) while bile acid concentrations of TCA (up to 200 μM) had no effect on the non-parenchymal cell fraction or on isolated cholangiocytes, whereas all of these cells had a cytokine response to LPS treatment. The induction of chemokines in hepatocytes was both dose- and time-dependent. Even at concentrations as low as 25 μM, albeit a pathophysiological relevant concentration, 24 h treatment of TCA significantly induced Ccl2 and Cxcl2 mRNA expression. Similar studies in human hepatocytes confirmed these observations. Further functional cell migration assays in transwell cultures, demonstrated that chemokines released into the culture medium from bile acid treated hepatocytes but not non-parenchymal cells were able to stimulate neutrophil chemotaxis. These observations confirmed that only hepatocytes but not other cells in the liver initiate an inflammatory response when exposed to pathophysiological relevant levels of bile acids.

To better understand why bile acids stimulated the expression of chemokines in hepatocytes but not other liver cells, we examined the role of the bile acid uptake transporter, Ntcp/Slc10a1. We found that bile acid induction of the chemokine Cxcl2 was significantly diminished when Ntcp expression was knocked down by siRNAs or when bile acid uptake was blocked by a newly synthesized Ntcp inhibitor in these cells. These findings indicated that bile acids had to be transported into the hepatocyte in order to initiate the synthesis of these cytokines. This conclusion is also consistent with observations from a rare case of an NTCP-deficient patient and also in *Ntcp*(*Slc10a1*)^−/−^ mice, where extremely high levels of bile acids were found in the plasma but both the patient and the *Ntcp* null mice were completely protected from cholestatic liver injury ^[17, 18]^. Because NTCP/Ntcp is a hepatocyte specific protein in humans and rodents, this explains why the other hepatic non-parenchymal cells are more resistant to bile acid induced tissue injury. These findings also argue against a role for a plasma membrane receptor in the hepatocyte in mediating this cascade of events ^[13]^.

To further investigate the intracellular events caused by bile acid accumulation in hepatocytes and also to better elucidate the signaling pathways involved in the induction of chemokines, we found that elevated hepatic levels of bile acid caused endoplasmic reticulum stress and mitochondria damage, evidenced by the organelle specific proteins leaking into cytosol as well as mitochondrial membrane potential changes after bile acid treatment, findings consistent with previous reports ^[19, 20]^. However, we did not detect ROS in these cells and the antioxidants N-Acetyl Cysteine or Mito-Q had little effect on repressing bile acid induction of Cxcl2. Alternatively, because bile acids caused mitochondrial damage, we speculated that toll-like receptor 9 (Tlr9) might be activated. As anticipated, bile acid induction of chemokine Cxcl2 was significantly diminished in *Tlr9*^−/−^ mouse hepatocytes. Similar results were also obtained in hepatocytes from the MyD88/Trif double knock out mouse, two proteins that are down-stream signaling molecules of Tlr9. Conversely, bile acids and the Tlr9 agonist synergistically stimulated Cxcl2 expression in mouse hepatocytes. Most importantly, liver injury was also reduced in hepatic specific Tlr9-deficient mice although not eliminated. Therefore, we speculate that there are likely to be additional signaling pathways involved in bile acid induction of chemokine expression in hepatocytes and further mechanistic studies will be needed.

Finally, to verify whether our findings from mouse cells and cholestatic rodent models described above were representative of the pathogenesis of cholestasis in human patients, we treated primary human hepatocytes with bile acids and also examined liver histology from patients with various cholestatic disorders. We found that GCDCA, the major endogenous bile acid in humans significantly induced chemokine CCL2 CCL15, CCL20, CXCL1 and IL-8 mRNA expression after exposure to 50 μM, a pathophysiological relevant concentration. We also found that serum ALT levels in cholestatic patients, measured around the time of obtaining liver tissue, positively correlated with the degree of periportal neutrophil infiltration in these biopsies. These findings are consistent with our rodent studies and emphasize the importance of the bile acid induced inflammatory response in the pathogenesis of cholestatic liver disease in man.

In summary, recent findings from our research and that of others indicate that a hepatocyte initiated inflammatory response plays a key role in bile acid induced cholestatic liver injury. Indeed, these studies point to several new targets for treatment of cholestasis since reducing the bile acid pool size, blocking bile acid uptake into hepatocytes, minimizing mitochondrial injury, and or blocking cytokine production or its inflammatory response are all distinct approaches that might reduce cholestatic liver injury. These findings emphasize that future therapeutic breakthroughs in this field may need to explore blocking multiple targets that are involved in the pathogenesis of this disorder as described in this report.

## Abbreviations

ALT: alanine aminotransferase
CCL: chemokine (C-C motif) ligand
CXCL: chemokine (C-X-C motif) ligand
Egr1: Early growth response protein 1
FACS: fluorescence-activated cell sorting
FXR: farnesoid X receptor
GCDCA: glycochenodeoxycholic acid
Mdr2: multidrug resistance protein 2
Ntcp: Na+-taurocholate cotransporting polypeptide
ROS: reactive oxygen species
TCA: taurocholic acid
Tlr9: toll -like receptor 9
